# Adenoid cystic carcinoma intermingled with ductal carcinoma of the breast: a case report and review of the literature

**DOI:** 10.1186/1752-1947-5-437

**Published:** 2011-09-06

**Authors:** Michael Kontos, Dimitrios Karles, Athanasios Petrou, Paraskevi TH Alexandrou

**Affiliations:** 1 First Department of Surgery, University of Athens, Laiko General Hospital, 17 Agiou Thoma Street, Athens 11527, Greece; 2 First Department of Pathology, University of Athens, 75 Mikras Asias Street, Athens 11527, Greece

## Abstract

**Introduction:**

Adenoid cystic cancer of the breast is a rare condition, and even rarer are the cases where it is histologically mixed with other variants of cancer within a single lesion. In this report, one of the few cases of mixed adenoid cystic breast cancer intermingled with the infiltrating ductal variant is presented. A subsequent review of the relevant literature presents the existing experience in treating mixed breast cancers with adenoid cystic components with regard to diagnosis, treatment, and prognosis.

**Case presentation:**

We describe a case of mixed adenoid cystic cancer of the breast with infiltrating ductal carcinoma in a 67-year-old Caucasian woman who underwent mastectomy with sentinel node biopsy.

**Conclusion:**

Surgery remains the cornerstone of treatment of these patients, and radiotherapy is administered when breast-conserving treatment is undertaken or a large tumor with affected lymph nodes is present. Hormonal treatment does not have a role, as estrogen receptors are always absent from both tumor components. Chemotherapy is nearly always administered on the basis of estrogen receptor and progesterone negativity and the more aggressive potential of the non-adenoid cystic component. The de-differentiation of an indolent type of cancer to a more aggressive one may affect the prognosis.

## Introduction

Adenoid cystic carcinoma (ACC) of the breast is a rare type of breast cancer, accounting for approximately 0.1% of all mammary tumors [1,2]. This histologic variant of cancer is commonly found in the salivary glands, where it bears an adverse prognosis. On the contrary, breast ACC usually presents a favorable course with infrequent lymph node involvement or distant metastases. The management of ACC is similar to that of more common breast cancer types and comprises breast-conserving treatment (BCT), mastectomy, full axillary clearance, sentinel node biopsy, chemotherapy, and radiotherapy.

Even rarer are the mixed tumors where breast ACC is histologically intermingled with infiltrating not otherwise specified (NOS) ductal carcinoma. We report a case of a 67-year-old woman with a tumor which proved to be a rare mammary neoplasia in which an infiltrative ductal NOS carcinoma merged with cribiform and solid areas of ACC.

### Case presentation

A 67-year-old Caucasian woman with an unremarkable medical and breast history was referred to our breast unit with a screen-detected right breast mass. Mammography had revealed a fairly well-circumscribed 2 cm lesion located deep and in close proximity to the nipple and was graded as M3 on the Breast Imaging Reporting and Data System scale. Her physical examination and ultrasound showed no abnormalities. A subsequent diagnostic biopsy revealed an infiltrating ductal carcinoma with an adenoid cystic component. The patient was treated with simple mastectomy and sentinel node biopsy. The histologic examination revealed a lesion of 1.7 cm maximum diameter with mixed infiltrating NOS ductal carcinoma and ACC characteristics. The four sentinel nodes were all free of cancer. She had a good post-operative course and remained well with no evidence of recurrence 24 months later.

The tumor was whitish tan and was ill defined in appearance and firm in consistency. No nipple or skin involvement was present. Both the nuclear grade of the lesion and the Bloom-Richardson grade were two based on the overall appearance of the tumor. The lymph nodes were negative. The tumor was staged as T1N0M0 and was estrogen receptor (ER)- and progesterone receptor (PR)-negative. The proliferative activity was low as measured by the Ki-67 labeling method (12%). Immunohistochemically, overexpression of the proto-oncogene *HER2*/*neu *was found in 15% of the carcinomatous cells. In fluorescence *in situ *hybridization analysis, which was performed at a later stage, no protein amplification was ascertained.

**Figure 1:**
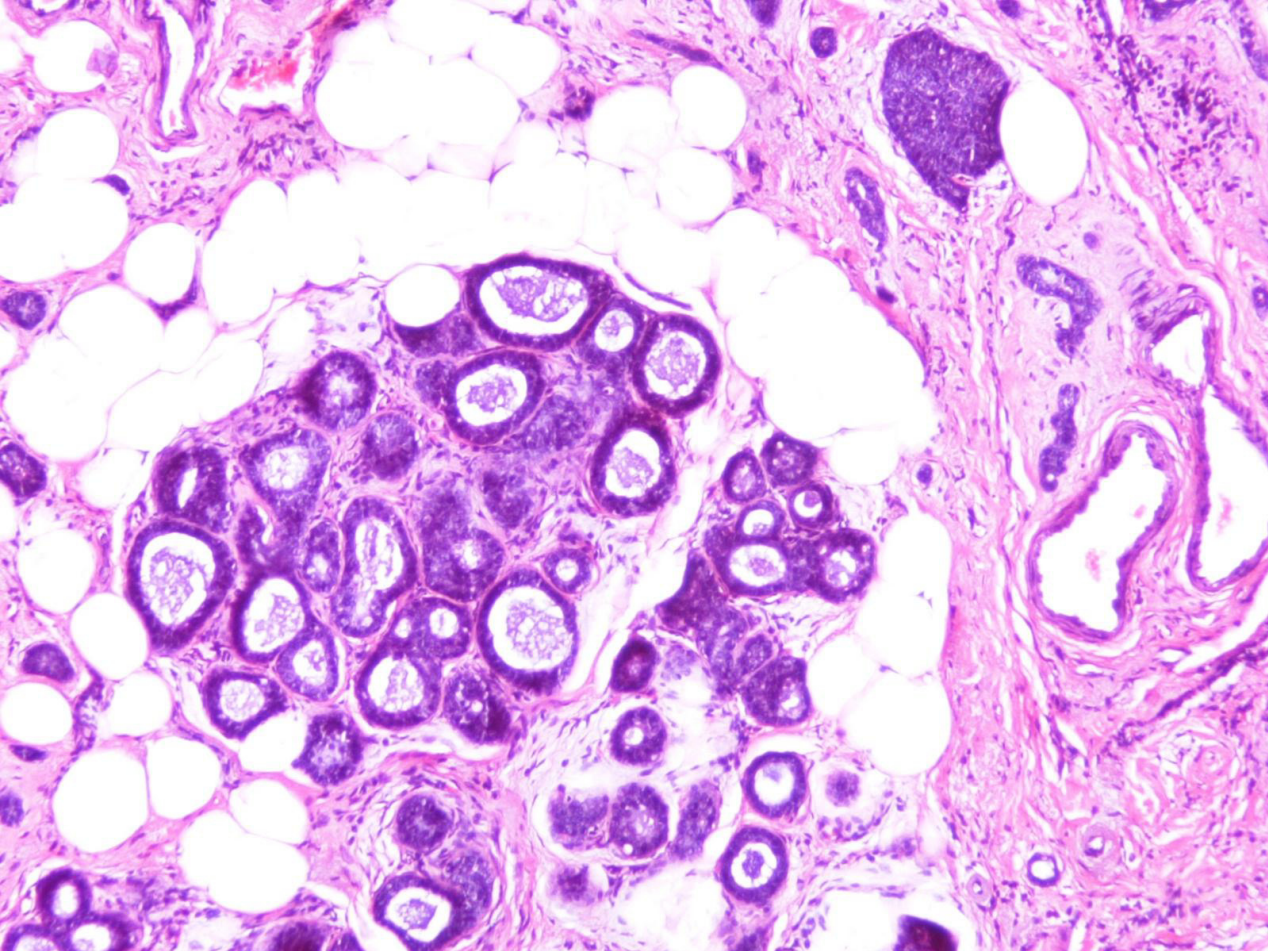
**Adenoid cystic and ductal neoplastic components intermingled each other (hematoxylin and eosin stain; original magnification × 200)**.

Two histological patterns were blending into each other without a clear-cut boundary between them (Figure 1). The dominant pattern, comprising more than 70%, was an ordinary, moderately differentiated ductal carcinoma NOS. It consisted of tubule islands and cribiform structures with epithelial cells exhibiting a high nuclear-to-cytoplasmic ratio, dark nuclear chromatin, and inconspicuous nucleoli. The mitotic index was low (< 2/10 hematopoietic tissue).

**Figure 2:**
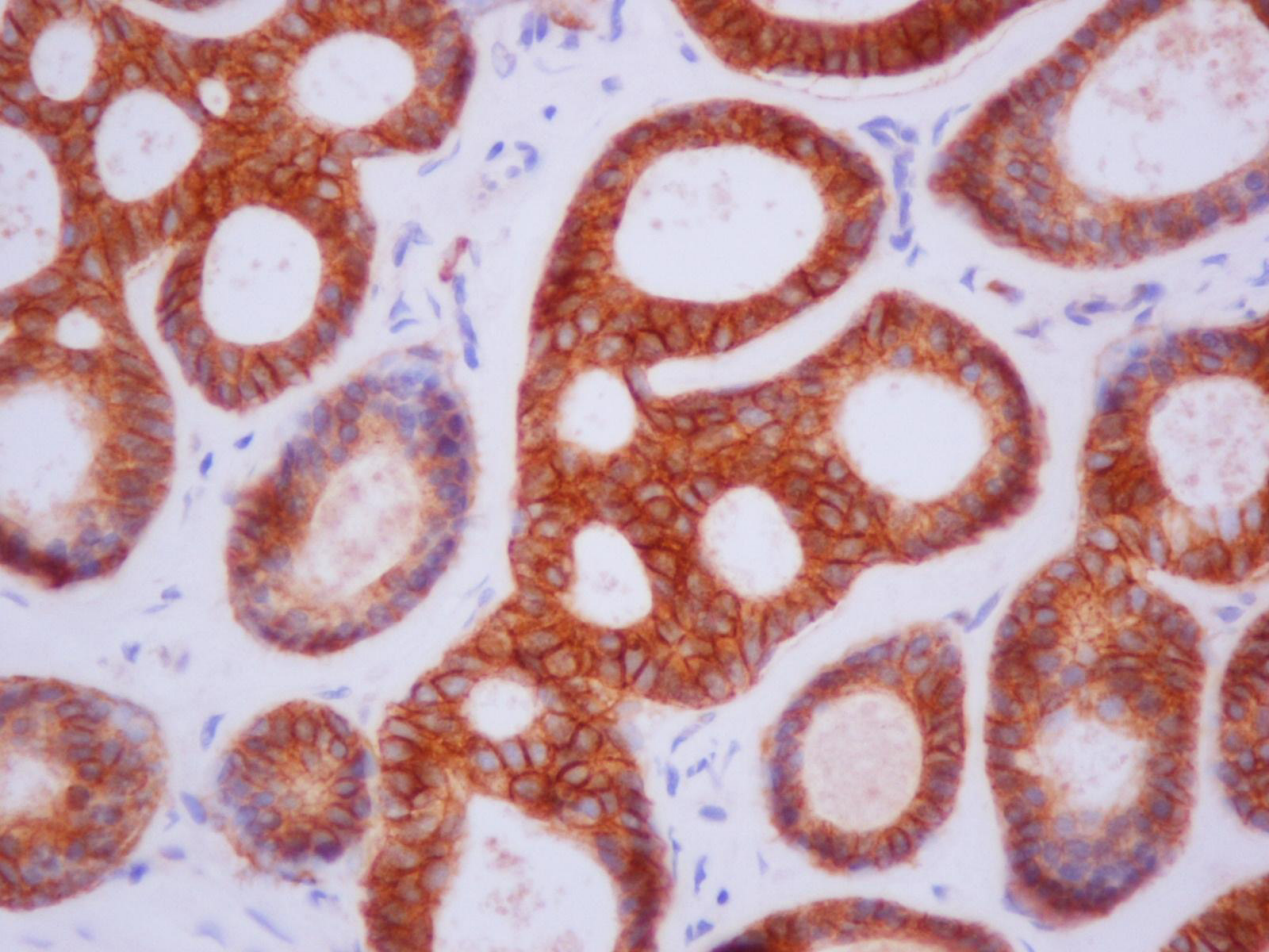
**Pseudo-cystic structures containing amorphous basement membrane material (hematoxylin and eosin stain; original magnification × 400)**.

**Figure 3:**
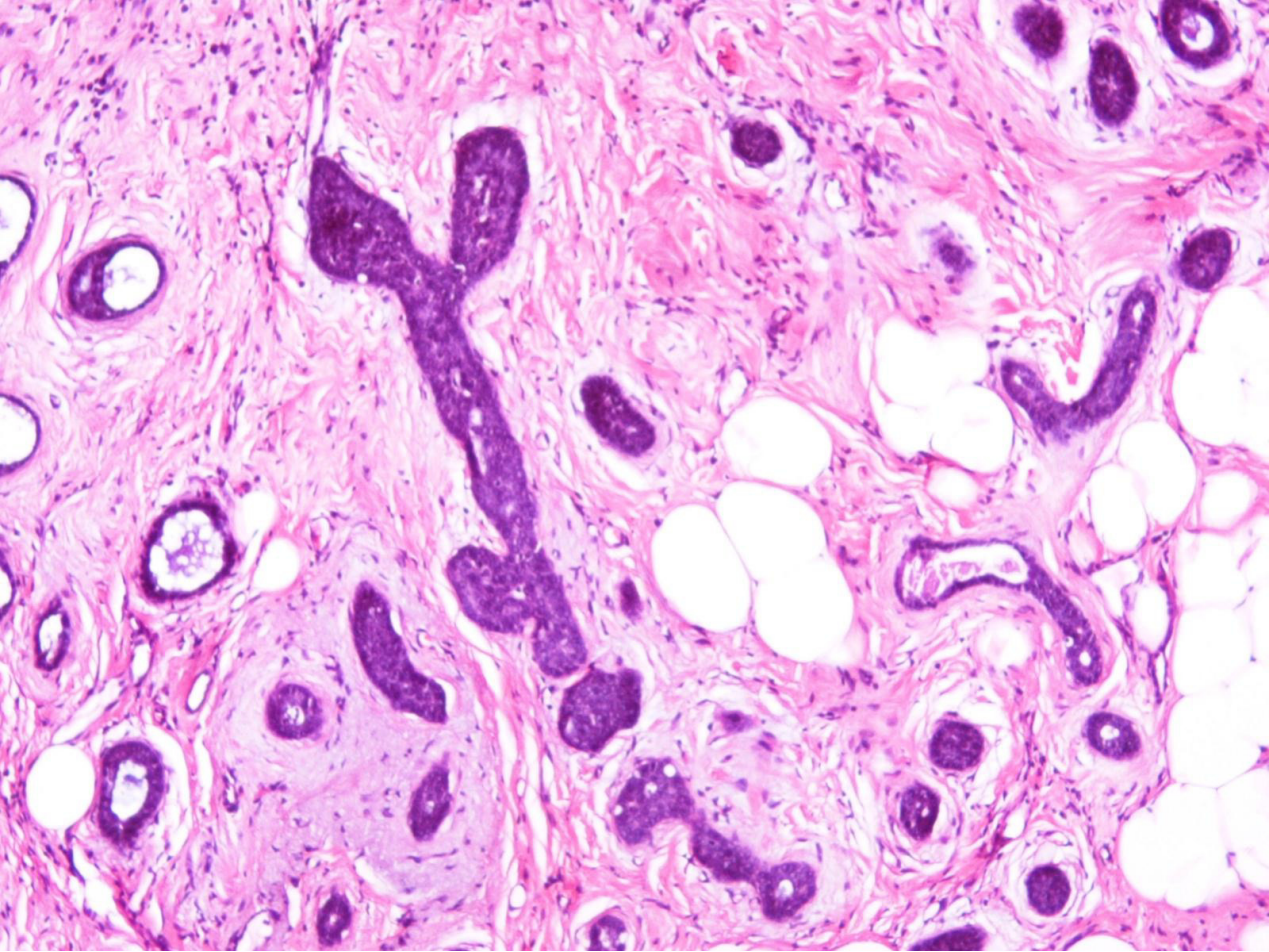
**Solid and tubular groups embedded in myxoid stroma (hematoxylin and eosin stain; original magnification × 400)**.

The rest of the histological pattern was limited and mainly consisted of well-defined nests and pseudo-glandular structures occasionally filled with homogeneous basophilic (periodic acid-Schiff stain-positive) material. Interestingly, the neoplasmic nests contained predominantly basaloid cells with sparse cytoplasm coexisting with epithelial cells with more abundant eosinophilic cytoplasm (Figures 2 and 3).

## Discussion

ACC of the breast is a rare histopathologic type of slow progression and represents only 0.1% of breast cancer cases. In sharp contrast to the extra-mammary counterpart, ACC of the breast has an excellent prognosis, as the incidence of lymph node metastasis is lower and distant metastases uncommon [1].

Even rarer are the cases of ACC intermingled with other types of breast cancer within a single lesion. Our case report is one of the few in the literature describing a patient with an ACC of the breast mixed with the ductal histological type. Furthermore, we present a review of the existing relevant cases of mixed breast cancers with an ACC component.

In the present study, the case of a 67-year-old woman with a non-palpable 1.7 cm right breast lesion is described. The radiologic evaluation was equivocal, but a mixed cancer with ACC and ductal components was found on biopsy. The patient agreed to proceed with a mastectomy (the patient's choice) and a sentinel node biopsy, which was negative. The hormone receptors were negative, and therefore there was no role for endocrine treatment.

Few cases of ACC have been described in the literature to date. Cabibi *et al. *[3] first reported an ACC intermingled with a small-cell carcinoma in a 40-year old woman. Mates *et al. *[4] later published a case report of a patient with bilateral breast cancer where the right side involved an ACC intermingled with a subtype of a ductal carcinoma (papillary). Another patient with ACC mixed with an "ordinary" invasive ductal and intra-ductal carcinoma was reported by Righi *et al. *[5]. Noske *et al. *[6] presented an ACC with spindle-cell carcinoma and melanoma (Table 1).

**Table 1:** Review of mixed ACC cases in the literature^a^

Study	Age, years	Histopathology	Surgery	Radiotherapy	Chemotherapy	ER/PR status
Mates *et al. *[4]	74	Micro-papillary invasive carcinoma mixed with ACC, 10 cm, N0	Mastectomy and full axillary clearance	No	Yes^b^	Negative/positive
Noske *et al. *[6]	51	Spindle-cell carcinoma, melanoma, and ACC, 2.5 cm, N0	Lumpectomy and sentinel node biopsy	Yes^c^	Yes^c^	Negative
Cabibi *et al. *[3]	40	Small-cell carcinoma with ACC, 2.5 cm, N0	Lumpectomy and full axillary clearance	Yes	Yes	Negative
Righi *et al. *[5]	63	Ductal and intra-ductal carcinoma with ACC, 4.0 cm, N1	Mastectomy and full axillary clearance	Yes	Yes	Negative (both ACC and ductal component)
Present study	67	Ductal carcinoma and ACC, 1.7 cm, N0	Mastectomy and sentinel node biopsy	No	No	Negative (both ACC and ductal component)

^a^ACC: adenoid cystic carcinoma; ER/PR: estrogen receptor/progesterone receptor. ^b^The patient had bi-lateral cancer (right side was mixed ACC and micro-papillary, and left side was lobular invasive breast cancer). ^c^Based on personal communication with A Noske.

The diagnosis may be challenging in mixed ACC cases, as some clinical and radiological features can be misleading. Furthermore, ACC of the breast is known for occasional demonstration of "benign" clinical or radiological characteristics [1]. Malignant lesions with ACC components can be well circumscribed on palpation, but careful radiologic evaluation and histopathology should set the diagnosis in experienced hands [3-6].

There is clearly no large experience with the treatment of mixed ACC of the breast. Surgical excision remains the cornerstone of treatment in operable cases, with two mastectomies and two cases of BCT reported in the literature [3-6]. Clear margins remain the desirable goal. A sentinel node biopsy was done in one case [6], and full axillary clearance was performed in three cases [3,4]. Radiotherapy has been applied after BCT [3,6] and after mastectomy with one affected node [5]. Chemotherapy was administered in three cases, while typically the ERs and PRs are negative. Chemotherapy is not routinely used to treat ACC of the breast; however, in mixed cases, it is likely that to be used on the basis of hormone receptor negativity and the more aggressive potential of the non-ACC component. The four previous reports of mixed ACC breast cancers and the present study are summarized in Table 1.

Regarding the histogenesis of these rare dual tumors, the hypothesis of de-differentiation prevails at the moment. Righi *et al. *[5] found that tumor morphology and immunohistochemical and clonality tests point toward the hypothesis that the two components are part of the same tumor and that part of the tumor underwent a progressive transformation, leading to the development of a more aggressive component. Cabibi *et al. *[3] surmised that the two different histological and immunohistochemical patterns might represent an example of de-differentiation along neuroendocrine phenotype lines occurring in a multi-potential neoplastic stem line already committed toward a myoepithelial phenotype. This de-differentiation can progress as far as a spindle-cell carcinoma or melanoma [6]. The de-differentiation of ACC to more aggressive types in salivary gland tumors was first reported by Cheuk *et al. *[7] in 1999. Nagao *et al. *[8] and Ide *et al. *[9] also reported similar cases for ACC located outside the breast.

Much of the clinical significance of discovering mixed ACC breast tumors lies in their prognostic information. The existence of a de-differentiated component of higher malignancy grade worsens the prognosis [7,8]. However, the exact magnitude of this is difficult to estimate because of the limited number of cases described in the literature and the wide diversity of the de-differentiation patterns. Another implication of mixed ACC breast cancers is the type of appropriate adjuvant treatment, as a chemotherapeutic agent or radiotherapy may not necessarily be effective for both components.

## Conclusion

At present, more questions than answers derive from the existing experience. Detailed publication of cases of such rare cancers will contribute to better understanding and more effective treatment. The current experience advocates surgery as the cornerstone of treatment, and radiotherapy is administered when BCT is undertaken or a large tumor with affected lymph nodes is present. As ERs are always absent from both tumor components, hormonal treatment does not have a role in ACC mixed tumors. Chemotherapy seems to have a significant role in the treatment of mixed ACC of the breast, not only due to the lack of hormone receptors but also because of the aggressiveness of the non-ACC component. Finally, de-differentiation of an indolent type of cancer (ACC) to a more aggressive one may affect the prognosis, although studies of more patients and longer follow-up are clearly needed.

## Consent

Written informed consent was obtained from the patient for publication of this case report and any accompanying images. A copy of the written consent is available for review by the Editor-in-Chief of this journal.

## Competing interests

The authors declare that they have no competing interests.

## Authors' contributions

MK was the main surgeon, had the scientific responsibility for the manuscript, and reviewed the draft extensively. DK was the assistant surgeon and composed the first drafts of the manuscript. PA performed the histological examination of the specimen and contributed to the writing of the manuscript. All authors read and approved the final manuscript.
